# Validation in Principal Components Analysis Applied to EEG Data

**DOI:** 10.1155/2014/413801

**Published:** 2014-09-08

**Authors:** João Carlos G. D. Costa, Paulo José G. Da-Silva, Renan Moritz V. R. Almeida, Antonio Fernando C. Infantosi

**Affiliations:** Biomedical Engineering Program, COPPE, Federal University of Rio de Janeiro, P.O. Box 68510, 21941-972 Rio de Janeiro, RJ, Brazil

## Abstract

The well-known multivariate technique Principal Components Analysis (PCA) is usually applied to a sample, and so component scores are subjected to sampling variability. However, few studies address their stability, an important topic when the sample size is small. This work presents three validation procedures applied to PCA, based on confidence regions generated by a variant of a nonparametric bootstrap called the partial bootstrap: (i) the assessment of PC scores variability by the spread and overlapping of “confidence regions” plotted around these scores; (ii) the use of the confidence regions centroids as a validation set; and (iii) the definition of the number of nontrivial axes to be retained for analysis. The methods were applied to EEG data collected during a postural control protocol with twenty-four volunteers. Two axes were retained for analysis, with 91.6% of explained variance. Results showed that the area of the confidence regions provided useful insights on the variability of scores and suggested that some subjects were not distinguishable from others, which was not evident from the principal planes. In addition, potential outliers, initially suggested by an analysis of the first principal plane, could not be confirmed by the confidence regions.

## 1. Introduction

A large number of variables is frequently required in many research fields and, especially, in the biomedical sciences. One of the most used methods for studying patterns in such large databases is the Principal Components Analysis (PCA) [1, 2]. PCA is suitable for dimensionality reduction and for exploratory purposes, allowing for the extraction of data features through variance maximization. However, as in any statistical model, a validation procedure must be employed if generalizability is required. Such procedures are even more important when only a small number of subjects/objects are available [3, 4]. Important statistics usually obtained in PCA are eigenvalues and principal component (PC) scores and, thus, nonparametric confidence intervals (C.I.) can be used to assess their variability. The latter can be, for example, generated by a resampling technique [4] and, then, computed as “confidence regions” around PC scores. Since the percent of explained variance is different according to each PCA dimension, corresponding C.I. are also different, helping outlier identification (longer intervals suggest extreme observations) [4].

One of these resampling techniques is the nonparametric bootstrap, in which samples are drawn with replacement in order to mimic the empirical probability function of the data [5]. Although visual cluttering may result, the bootstrap (BST) can be employed for defining confidence regions in PCA, thus helping graphical display interpretation [4]. However, few texts address the subject of PCA confidence regions derived from BST. One of them is Linting et al. [6], in which 90% BST ellipses were drawn for a nonlinear PCA used to study interactions between children and caregivers in nonmaternal child care. By comparing the results with those from a linear PCA, they suggested a guideline for users who wish to employ the BST procedure in linear and nonlinear PCA.

Classification of electroencephalographic (EEG) signals is an objective of many neurological studies, for example, for staging a neurologic disease or for brain-computer interface (BCI) systems. These systems, briefly, concern the transformation of human thoughts (through acquired EEG signals) into a computer system, for instance, for helping people with motor or spelling impairments during specific tasks [7]. The electroencephalogram is the registry of a spatial-temporal cortical activity recorded from electrodes spatially placed on the scalp region and is mainly characterized by signals with different frequency bands, such as theta (4–8 Hz), alpha (8–13 Hz), and beta (13–30 Hz), and amplitude varying with pathological conditions and in specific behavior states (e.g., sleep or vigil, eyes open or closed) [8, 9]. Although online classification tasks are a prerequisite for practical BCI purposes, extensive offline studies are needed before establishing a trustworthy BCI device, hence indicating the importance of validation procedures.

The aim of this paper is to present three validation procedures for PCA using the nonparametric bootstrap, with an application to EEG data. These procedures allow for assessing the sampling variability of PC scores and the number of axes to be retained for analysis, especially if only a small number of subjects are available. The method concerns plotting “confidence regions” and constructing a “validation set” for PC scores (the centroids of the confidence regions). A variant of the ordinary nonparametric bootstrap called the partial bootstrap (PBST) was used to this end. Furthermore, a validation procedure was employed in order to confirm the number of nontrivial axes to be analyzed. The assessment of sampling variability of the PC scores was performed through the areas of the confidence regions, while the centroids were compared to the original scores through an unsupervised classification algorithm. An example with correlated attributes derived from time and frequency-domain EEG signals was used for introducing the proposed approach. The theory of PCA and nonparametric BST is introduced in Sections 2 and 3, respectively, while the validation methods are presented in Section 4. In Section 5, the method is applied to EEG data obtained from a postural control protocol.

## 2. Principal Components Analysis

Principal Components Analysis is comprehensively presented in many multivariate statistics textbooks, such as Jolliffe [2] and Lebart et al. [10], and only a brief introduction is given here. From *p* variables observed on *n* objects (an *n* × *p* matrix), that is, a raw data matrix **X**, PCA derives new variables as linear combinations of the original ones, defined from a new orthogonal coordinate system onto which the original space is projected. This new system summarizes the total data variation in decreasing order so that the first new variable has the largest variation, the second has the second largest, and so on. These new variables are the principal components. The singular value decomposition (SVD) is used to estimate this new orthogonal space, by factoring **X** as [11]

(1) $$\begin{array}{c} X=U\times D\times V^{T}, \end{array}$$

where **U** and **V** are the left and right singular vectors matrices, respectively, **U** **U** ^**T**^ = **I**_*n*_(*n* × *n*), **V** ^**T**^ **V** = **I**_*p*_(*p* × *p*), and the superscript **T** indicates the transpose of the matrix. **D** is a diagonal matrix with singular values *λ*_*i*_ in decreasing order *λ*_1_ ≥ *λ*_2_ ≥ ⋯≥*λ*_*p*_ ≥ 0, *i* = 1,2,…, *p*. Squaring **D** and dividing it by (*n* − 1), one obtains

(2) $$\begin{array}{c} \text{cor}(X)=V\times \Sigma \times V^{T}, \end{array}$$

where cor(**X**) is the sample correlation matrix if **X** has standardized variables. Matrix Σ is also a diagonal matrix, with elements related to the variance of **X**, in which *σ*_1_^2^ ≥ *σ*_2_^2^ ≥ ⋯≥*σ*_*p*_^2^ ≥ 0, so that (*n* − 1)*σ*_*j*_^2^ = *λ*_*j*_^2^,  *j* = 1,2,…, *p*.

The PC scores (**Z**) are obtained as follows:

(3) $$\begin{array}{c} Z=X\times V=U\times D. \end{array}$$

Therefore, elements of **Z** are linear combinations of the elements of **X**, with component coefficients given by the column-vector of **V** (which is called the* loadings* matrix). The matrix **V** defines, thereby, an orthonormal basis and its columns are linearly independent vectors. Indeed, **Z** is the projection of **X** onto the orthonormal basis **V**.

As mentioned, of the most common uses of PCA is the dimensionality reduction of **X**, keeping as much information (variance) as possible. If the reduced dimensionality is *m* ≤ *p*, one may consider the model (in elementwise representation):

(4) $$\begin{array}{c} x_{ij}=\overset{m}{\underset{t=1}{\sum }}u_{it}\lambda_{t}v_{tj}+\varepsilon_{ij}, \end{array}$$

where *u*_*it*_ and *v*_*tj*_ are the elements of matrices **U** and **V**, respectively, while *ε*_*ij*_ represents the residual terms or the noise present in the data, *i* = 1,2,…, *n* and *j* = 1,2,…, *p*. The proportion of variance explained by each dimension up to dimension *m* is given by

(5) $$\begin{array}{c} \text{var}\%=\frac{\sum_{j=1}^{m}\lambda_{j}}{\sum_{j=1}^{p}\lambda_{j}}\times 100\%. \end{array}$$

The procedure for choosing the number of principal components *m* to be retained is not well-defined. One method is the Scree plot, based on a plot of eigenvalues against their order [12, 13]. Some authors suggest other empirical methods such as the retention of a number of dimensions corresponding to a fixed proportion of explained variance (usually 70–90%) and the Kaiser's rule (retaining the eigenvalues of the correlation matrix higher than unity) [2, page 113–115] and [12, 13]. Statistical approaches have also been proposed, such as the Bartlett's test or eigenvalues bootstrapping [13]. However, as Jolliffe [2, page 133] pointed out, there is still no clear advantage of a specific method over the others.

## 3. Nonparametric Bootstrap

The nonparametric BST is a computer-intensive technique, which attempts to replicate the probability distribution of a statistic of interest by resampling with replacement from the original sample (the observed data) a predefined (*R*) number of times [5]. Usually, this procedure generates new samples of the same size *n* of the original one, providing a mathematical framework for inferring the statistical accuracy of the desired estimate [14]. Thus, in summary, the statistic of interest ($\tilde{\theta }$) is the observed value of some unknown population parameter *θ*, and the nonparametric BST generates *R* replicated samples of the original data (the BST samples), resulting in the set ${\tilde{\theta }}_{\text{set}}^{*}=\{{\tilde{\theta }}_{1}^{*},{\tilde{\theta }}_{2}^{*},\ldots ,{\tilde{\theta }}_{R}^{*}\}$. If the observed data is independent and identically distributed, the BST estimate of the observed value (${\tilde{\theta }}^{*}$) can be calculated from ${\tilde{\theta }}_{\text{set}}^{*}$ as

(6) $$\begin{array}{c} {\tilde{\theta }}^{*}=\frac{\sum_{r=1}^{R}\theta_{b}^{*}}{R}, \end{array}$$

implying that ${\tilde{\theta }}^{*}$ is an estimate of the true value *θ*. The accuracy of the BST estimates can be represented by confidence intervals (C.I.) calculated from ${\tilde{\theta }}_{\text{set}}^{*}$. The percentile method [14] is the simplest method for BST C.I. and is based on the percentiles of ${\tilde{\theta }}_{\text{set}}^{*}$, as

(7) $$\begin{array}{c} \text{C.I.}=[{\tilde{\theta }}_{\text{set}}^{*}(\frac{\alpha }{2}),{\tilde{\theta }}_{\text{set}}^{*}(1-\frac{\alpha }{2})], \end{array}$$

where ${\tilde{\theta }}_{\text{set}}^{*}(\alpha /2)$ and ${\tilde{\theta }}_{\text{set}}^{*}(1-\alpha /2)$ are the 100*α*/2% and 100(1 − *α*/2)% percentiles of the ${\tilde{\theta }}_{\text{set}}^{*}$ and *α* is the desired confidence level. For example, for *α* = 0.05 and *R* = 1000, the C.I. inferior/superior limits are the 24th/976th elements of ${\tilde{\theta }}_{\text{set}}^{*}$. In general, it is advisable to have a large *R* [14], and, for PCA, Lebart [4] advocates *R* ≥ 30, while Diaconis and Efron [15] employed 100 replications in a PCA study for grading college students.

## 4. Validation and Stability

The performance of a model is always better on data on which the model was estimated, and this rule applies for both exploratory and predictive methods [3, 16]. In order to evaluate the results obtained by the whole (or part of) observed sample (the training set), the model can be applied to a different data (the validation set), assessing its generalization ability [17, 18]. The procedure of applying the obtained model to new data is usually called* validation*. Therefore, modeling demands rigorous validation procedures, since a good model is supposed to have generalizability [16]. Basically, there are three kinds of validation: internal, external, and relative, the first being most commonly used due to its simplicity and lower costs. In the internal validation, the observed data can be split in two or more sets (such as cross-validation) or BST methods (one for training and the others for validating the model), while in the external validation, a new but plausible dataset is presented to the model. For the relative validation, a different model is applied to the available data. When the training set has a small number of subjects, BST becomes a good option for internal validation, since all subjects can be used for model development (no observation is discarded), and BST samples can be used for validation. Furthermore, the generalization concept described above can be connected to the concept of stability in PCA, because if the score coordinates do not change markedly, their positions onto principal planes can also be considered stable.

Applying nonparametric BST to, for instance, the above defined matrix **X**(*n* × *p*), different matrices may be generated by the replication of different rows, and their singular values and singular vectors will no longer be the same. The SVD applied to each of the *R* BST matrices is

(8) $$\begin{array}{c} X_{r}^{*}=U_{r}^{*}\times D_{r}^{*}\times V_{r}^{*T}, \end{array}$$

where ∗ denotes a BST sample and *r* = 1,2,…, *R*. **D**_r_* has singular values in decreasing order *λ*_1r_* ≥ *λ*_2r_* ≥ ⋯≥*λ*_pr_* ≥ 0, and Σ_r_* = **D**_*r*_^∗2^/(*n* − 1). Through these concepts, validation procedures using nonparametric BST can be applied to PCA.

### 4.1. Assessing the Number of Nontrivial Axes

If the chosen dimensionality is m* < *p*, no overlapping between BST eigenvalues for (*α* = 0) will occur if

(9) $$\begin{array}{c} \min(\sigma_{1}^{*})-\max(\sigma_{2}^{*})>0,\\ \min(\sigma_{2}^{*})-\max(\sigma_{3}^{*})>0,\\ \vdots \\ \min(\sigma_{m^{*}-1}^{*})-\max(\sigma_{m^{*}}^{*})>0, \end{array}$$

where *σ*_k_* = {*σ*_*k*1_*, *σ*_*k*2_*,…, *σ*_kR_*}, for *k* = 1,2,…, m*; thus the number m* obtained from BST can be compared to the number *m* obtained by Scree plot.

### 4.2. Assessing the Variability of PC Scores

After the application of BST to **X**, *R* replicated matrices are obtained. Due to their different axes (defined by different eigenvectors), they cannot be directly compared to the original space (defined by the eigenvectors of the original correlation matrix), because of axes reflection or inversion [4, 19]. Since replicated samples do not have necessarily the same subjects compared to those in the original sample, different eigenvalues and eigenvectors can occur, and a correction procedure is needed, such as that provided by Procrustes Analysis [20]. To circumvent this problem, the PBST can be applied, consisting of projecting replicated components scores (as “supplementary” points) onto the orthonormal matrix **V**:

(10) $$\begin{array}{c} {\overset{⌢}{z}}_{ir}=x_{ir}^{*}\times V_{}^{}, \end{array}$$

where ${\overset{⌢}{z}}_{ir}$ is the *i*th component score of the *r* replicated, standardized object (**x**_ir_*). Therefore, *nR* object scores can be visualized in the original space, generating *n* clouds of points. This approach has the advantage of maintaining the original PC planes, which is a better estimate than any of the replicated planes [4]. Thus, (10) can be expressed as

(11) $$\begin{array}{c} {\overset{⌢}{Z}}_{\text{parc}}=X_{}^{*}\times V_{}^{}, \end{array}$$

where

(12) $$\begin{array}{c} {\overset{⌢}{X}}^{*}=[\begin{array}{c} X_{1}^{*}\\ X_{2}^{*}\\ \vdots \\ X_{R}^{*} \end{array}], \end{array}$$

and **V** is limited to *m* dimensions (*m* × *m*) after the dimensionality reduction procedure is applied.

Since PCA displays are usually shown in a low-dimensional space, confidence regions are represented as polytopes [21] or, in a two-dimensional space, as polygons (or convex hulls) [22]. The interpretation of these polygons basically takes into account overlapping (which suggests similar objects) and spread (widespread polygons suggest unstable score coordinates, while narrow polygons suggest stability). Furthermore, these confidence regions allow for the estimation of new PC scores (through their centroids). Although any value of *α* can be used, Efron [23] states that *α* = 0.10 is satisfactory in most cases, while Lebart [4] pointed out that when *α* = 0 untypical values (e.g., outliers) can be easily identified (through the longer edges of the plotted convex hull).

### 4.3. Validation of PC Score Coordinates

The area (in square units) of a polygon can be calculated as

(13) $$\begin{array}{c} S=\frac{1}{2}[|\begin{array}{cc} \begin{array}{c} x_{1}\\ y_{1} \end{array} & \begin{array}{c} x_{2}\\ y_{2} \end{array} \end{array}+|\begin{array}{cc} \begin{array}{c} x_{2}\\ y_{2} \end{array} & \begin{array}{c} x_{3}\\ y_{3} \end{array} \end{array}+\cdots +|\begin{array}{cc} \begin{array}{c} x_{l}\\ y_{l} \end{array} & \begin{array}{c} x_{1}\\ y_{1} \end{array} \end{array}], \end{array}$$

where (*x*_1_, *y*_1_), (*x*_2_, *y*_2_),…, (*x*_*l*_, *y*_*l*_) are the *l* vertices' coordinates of the polygon, in clockwise order, and |·| is the determinant of the matrix. Absolute value can be calculated, if necessary, and the centroid coordinates $\left({\overset{-}{x}}_{c},{\overset{-}{y}}_{c}\right)$ calculated from any polygon are given by

(14) x−c=16S·∑i=1l(xi−1+xi)(xi−1yi−xiyi−1),y−c=16S·∑i=1l(yi−1+yi)(xi−1yi−xiyi−1(.

Thus, the centroid can be considered as the BST estimate (BST centroids) of the true component score.

The BST centroids are, therefore, estimates of the PC score coordinates, and the comparison of original scores and BST centroids allows for the comparison of both models, using, for example, an unsupervised classification method. These clustering methods concern procedures where the groups are not known a priori and the researcher must choose, based on previous knowledge or on some criteria, the number of clusters present in the data. This subjective procedure is mainly employed to visualize or suggest clusters, generating hypothesis for later investigation [24].

One kind of unsupervised classification method is the hierarchical algorithm, in which a nested-tree diagram (the dendrogram) is generated, suggesting, by inspection, the underlying clustering structure of the data. There are, basically, two kinds of hierarchical classification algorithms, the divisive and the agglomerative, which group objects according to some clustering rule [25]. Agglomerative Hierarchical Algorithms (AHA) are some of the most used classification algorithms and start by grouping two objects into a single cluster, and at each step of the algorithm, new objects are aggregated, forming a new cluster, and so on, until, in the last step, all objects are joined into a single cluster. “Cutting the tree” at some distance is one of the procedures for defining the cluster structure in AHA [24, Section 3.3.2], and the Average Linkage Algorithm is considered the most stable AHA [26]. Therefore, an AHA using this method can be used to compare groups generated by ordinary PC scores and BST centroids.

## 5. Application

### 5.1. Subjects

A data set from a postural control protocol was used in this study, including stabilometric and EEG signals. Thirty-one subjects (21 males and 10 females), ages 21 to 45 (31.0 ± 6.6) years, height 154 to 187 (172.7 ± 9.4) cm, and body weight 46 to 107 (73.3 ± 17.3) kg, participated in the initial study. All subjects presented no history of neurological pathologies, osseous, muscles or joints diseases, or equilibrium disorders. An anamnesis was performed to obtain information about headaches, illnesses, vertigo, eyestrain, and the use of contact lens or glasses. Subjects using lens or glasses were included when no problem with their use was reported. The study was approved by a Local Institutional Review Board (IESC/UFRJ - Ref. 100/2011). None of the authors participated as a volunteer.

### 5.2. Experimental Protocol

**Figure 1:**
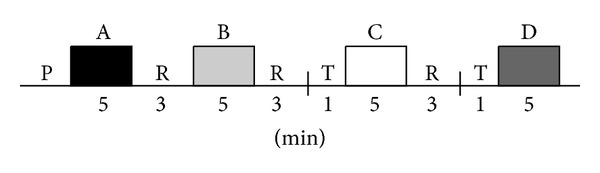
Complete experimental protocol sequence for the acquisition of EEG signals in 31 subjects (21 males and 10 females). P indicates the preparation procedure; R refers to the resting interval of three minutes; T indicates the transition between sat and upright standing positions.

The EEG and stabilometric signals were acquired simultaneously, but only EEG signals were analyzed here. The experiments were performed in an electromagnetically shielded room, under controlled environmental conditions (23°C, attenuated sound and light control), with the subject barefooted on a force platform. The feet position (angle: 30°; heels 2 cm apart) was previously delineated to standardize the same support base during the tests. The EEG signals were acquired during five minutes, with the subject in distinct postural conditions: (i) resting in a comfortable armchair with eyes closed (spontaneous EEG with room lights off, denoted as “A”); (ii) the same position as (i), but with eyes open (“B”); (iii) during stabilometric test in upright standing position with eyes open (denoted as “C”); and (iv) eyes closed (“D”). The trials with eyes open condition were conducted with room lights on and with the subject watching a white wall located 1 meter apart from the force platform. An interval of three minutes was taken between each condition, and the subject remained seated in the chair during this period. The stabilometric tests were performed one minute after the subject was standing on the force platform, in order to allow for the recovery of balance after rising from the chair.  Figure 1 shows the experimental protocol sequence.

The EEG recordings were acquired using the BrainNet—BNT 36 (EMSA, Brazil, http://www.emsamed.com.br) device at a sampling frequency of 400 Hz and 16 bit A-to-D precision, with electrode position according to the International 10/20 System (monopolar derivations, averaged bilateral earlobe reference and ground in FPz). Scalp electrode impedances were below 5 kΩ throughout the session. The EEG recordings were analog-filtered by a fourth-order low-pass Butterworth with cutoff frequency at 100 Hz (antialiasing) and second-order high-pass Butterworth at 0.1 Hz and also by a digital notch filter in 60 Hz. The power spectral densities (PSDs) were determined by an additional offline digital filtering using a fourth-order, forward-reverse band-pass (1–40 Hz), Butterworth filter [27].

### 5.3. Data

The complete data set consisted of 5-minute EEG recordings (O1, O2, P3, P4, C3, C4, T3, T4, T5, T6, F3, F4, F7, F8, Fp1, Fp2, Fz, Cz, Pz, and Oz derivations) for the conditions A, B, C, and D. In order to reduce display cluttering, only one occipital derivation (O1) was used in this study. The O1 EEG signals were first segmented into 1-second zeroed-mean epochs of 400 samples. An artifacts rejection methodology proposed by Simpson et al. [28] was also applied, resulting in a different number of epochs for each volunteer and condition (min⁡ = 20, max⁡ = 300). To allow for better precision of estimates and for computational convenience (all epochs were stored in an array) only those volunteers with a minimum of 150 free artifacts epochs were retained in the study (24 subjects).

### 5.4. Variables in the Frequency and Time Domain

A rectangular window was subsequently applied to each epoch, and the averaged periodogram was calculated by the Bartlett method. Six variables were extracted from the periodogram: maximum of the PSD magnitude of alpha (8–13 Hz), theta (4–8 Hz), and beta (13–30 Hz) bands in log⁡10 (micro V^2^/Hz) and an estimate of alpha, theta, and beta band power, defined as the trapezoidal area centered in the maximum peak of respective bands log⁡10 (micro V^2^).

For each epoch, four statistics were estimated: the root mean square (RMS), the difference between maximal positive and minimal negative values (Mm); the standard deviation of the samples (SD); and the skewness coefficient [29]. Then, the median of each statistic for all 150 epochs was determined.

### 5.5. Variable Statistics

**Figure 2:**
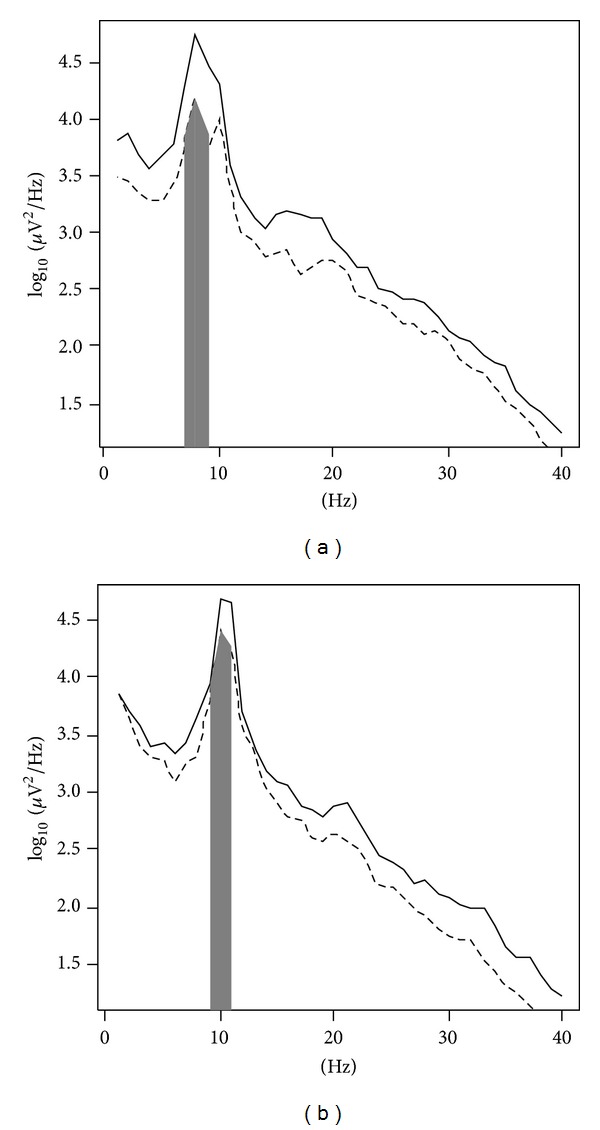
PSD of volunteers 1 (a) and 3 (b), solid line for condition A, and dashed line for condition B. (a) Maximum for condition A at 4.75 and maximum for condition B at 4.17. (b) Maximum for condition A at 4.68 and maximum for condition B at 4.40. Power for condition B was calculated as the area of the highlighted region in grey.

Mean ± standard deviation for frequency domain variables was 6.75 ± 1.26 (alpha band power, range 4.28–9.12), 5.44 ± 0.73 (beta band power, range 4.11–6.82), 6.12 ± 0.96 (theta band power, range 4.54–9.12), 3.52 ± 0.68 (alpha band maximum, range 2.24–4.75), 2.72 ± 0.36 (beta band peak, range 2.03–3.37), and 3.07 ± 0.50 (theta band maximum, range 2.27–4.75). The Shapiro-Wilk test suggested that the alpha and beta band power (log⁡10) and the alpha and beta band maximum (log⁡10) variables were Gaussian. In Figure 2, PSDs of two volunteers (conditions A and B) are shown, with the areas corresponding to power at distinct bands highlighted. For volunteer 1, maximum peak for eyes open was achieved at 8 Hz, a transition frequency between theta and alpha bands (Figure 2(a)), while for volunteer 3, the maximum peak in the same condition occurred at 10 Hz (Figure 2(b)). For time domain variables, values were 12 ± 6 (RMS, range 4–30), 12 ± 6 (SD, range 4–30), 0.1 ± 0.1 (skewness, range −0.1–+0.3), and 57 ± 26 (Mm, range 22–137). There were positive and significant (*P* ≪ 0.001) correlations (rho's Spearman coefficient, range 0.62–1.00) between all variables, with perfect correlation (rho = 1.00) between alpha power and alpha maximum and between RMS and SD (redundant variables).

### 5.6. PCA and Nonparametric BST

Considering the 24 subjects to whom the experimental protocol was applied (conditions A and B) and the extracted variables from the EEG signal (six in frequency and four in time domains), the resulting data matrix (**X**) with 48 rows and 10 columns (variables) was constructed. The SVD algorithm was applied to the zeroed-mean, standardized data matrix, and the **U**, **D**, **V**, and **Z** matrices were calculated, according to (1) and (3). The number of axes to be retained was assessed by the Scree plot and validated through an analysis of the nonoverlapping confidence intervals (*α* = 0) of the replicated eigenvalues.

The nonparametric BST was performed according to the following steps.

1. Resampling the rows (of **X**) with replacement, with *R* = 1000, which resulted in *R* replicated matrices 48 × 10.
2. The *R* matrices were mean-centered and standardized.
3. The SVD algorithm was applied to all *R* standardized matrices.
4. **Z**_par_ = X* × **V** was obtained by (11).
5. The extreme points of all 48 clouds were determined.
6. The convex hulls (confidence polygons) were plotted around each object, with *α* = 0 to analyse likely outliers.
7. The BST centroids and the areas of confidence polygons were calculated according to (14) and (13), respectively.

### 5.7. Validation

Validation was carried out as described in Section 4. To assess the variability of the PC scores, the areas of their corresponding convex hulls were compared, while BST centroids were compared to original scores by the dendrograms originated from an AHA, average method. The number of retained axes was assessed by the 100% C.I. obtained by a nonparametric BST.

The significance level adopted was 5% and data processing used the open access *R* statistical software [30], packages* R.matlab* [31],* signal* [32],* e1071* [33], and* pracma* [34]. Convex hulls and confidence polygons are terms interchangeably used in this text. Spearman correlation coefficient and Shapiro-Wilk tests were applied to verify correlation and Gaussianity, respectively.

### 5.8. Results

**Figure 3:**
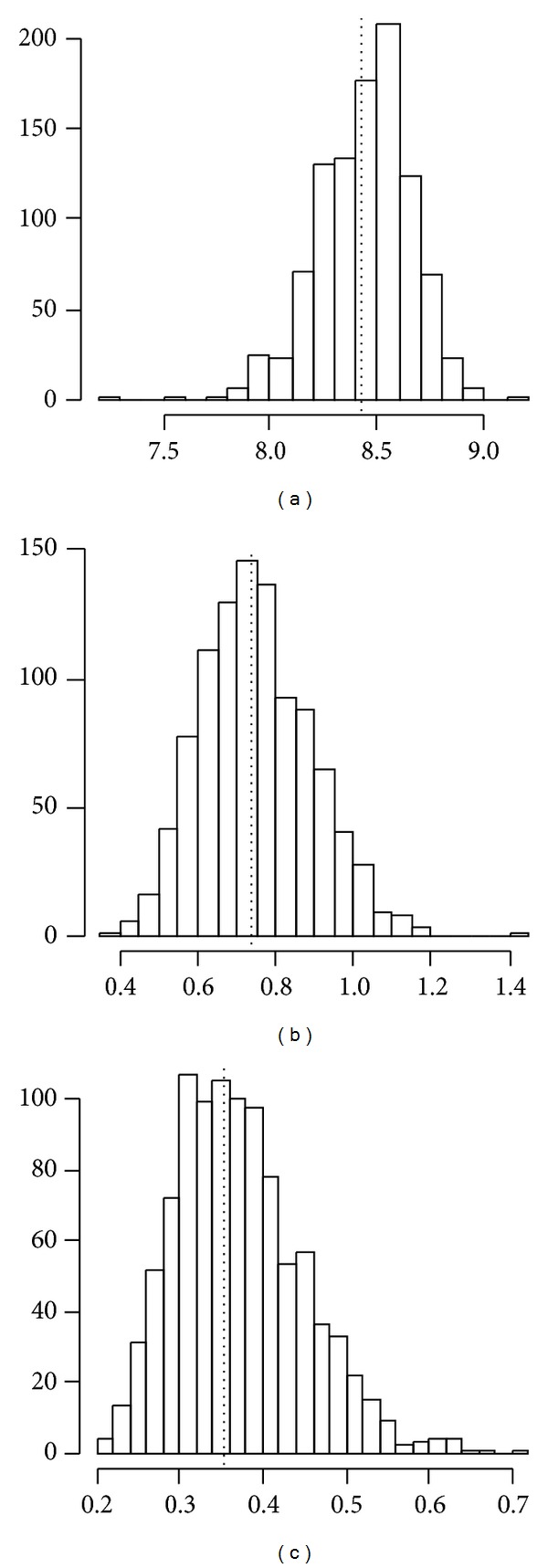
Histogram of replicated eigenvalues. (a) First eigenvalues; (b) second eigenvalues; (c) third eigenvalues. An overlapping between the 100% C.I. of second and third eigenvalues can be seen.

The dimensionality suggested by the Scree plot was two, corresponding to 91.6% of the explained variance (1st eigenvalue: 8.42; 2nd eigenvalue: 0.74). The coefficients for these two PCs are shown in Table 1. The dimensionality analysis was also confirmed by the C.I. of replicated eigenvalues (Figure 3).

Since the first PC is a linear combination with almost equal weights, none of these variables can be said to be “more influential.” Therefore, in this component, signal scores contrast only in relation to the origin. The second PC, however, shows a contrast between the beta and theta bands.

The histograms for the first three replicated eigenvalues are shown in Figure 3, with original eigenvalues in dashed lines. No overlapping between the first and two replicated eigenvalues occurred, since min⁡(*σ*_1_* = 7.30) > max⁡(*σ*_2_* = 1.40). On the contrary, overlapping is present in the C.I. of the second and third replicated eigenvalues (Figures 3(b) and 3(c)). Explained variances in replicated samples, in two dimensions, varied from 86.1% to 95.0% (mean = 91.9%).

**Figure 4:**
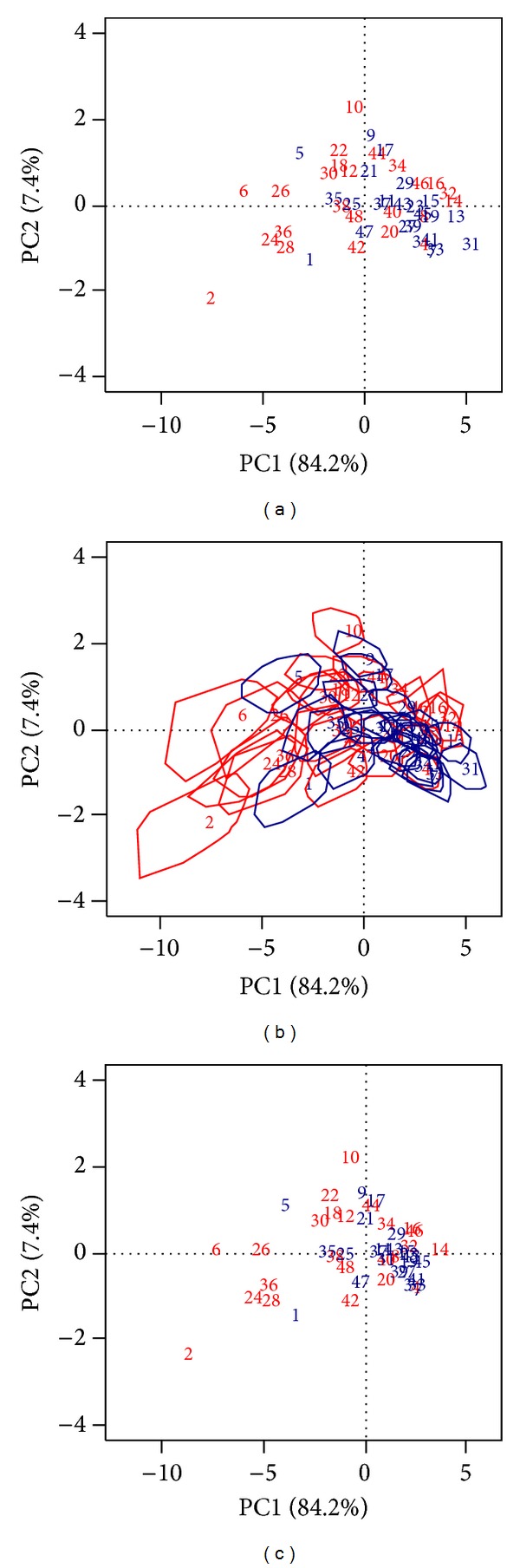
(a) Principal plane for signal scores, numbers from 1 to 48; (b) PC scores surrounded by their corresponding convex hulls; (c) BST-generated centroids. Signals corresponding to A and B conditions in red and blue, respectively.

The projection of all replicated matrices in the original orthonormal basis provided 48000 points, which were synthetized by the convex hulls encompassing all 1000 replicated samples for each score. The number of replications varied between 931 (subject 17) and 1077 (subject 24), with mean = 1000. The principal plane is depicted in Figure 4(a), where the first PC has 84.4% of explained variance, while, in Figure 4(b), convex hulls are drawn around the original coordinates of the 48 scores. Extended overlapping convex hulls suggest signals with similar characteristics; therefore, their BST centroids are closer in the display. The area of the convex hulls varied between 0.96 and 7.79 (mean = 2.25). The two largest areas correspond to signals 6 (7.79) and 2 (7.37), while the smallest areas correspond to signals 29 (0.96) and 23 (1.02). Since these areas are located on opposite sides of the first PC, it can be said that this PC also discriminates between larger and smaller areas.

The areas of signals number 2 and number 6 also deserve attention. The convex hull corresponding to the latter is placed onto the second and third quadrants, while the former is placed on the third one. Signal 2 (female; Figure 3(a); solid line) had the highest and coincident measures for alpha (9.12) and theta (9.12) power and for alpha (4.75) and theta (4.75) maximum (maximum peak was at 8 Hz in the transition frequency between alpha and theta bands). Also, this subject had the highest RMS (30.1), SD (30.1), skewness (0.31), and Mm (136.7). Signal 6 (male; Figure 3(b); solid line) had the highest values of beta power (6.82) and maximum beta (3.37). An analysis on overlapping polygons revealed a similarity for other signals, for each side of the first PC. The BST centroids are shown in Figure 4(c).

**Figure 5:**
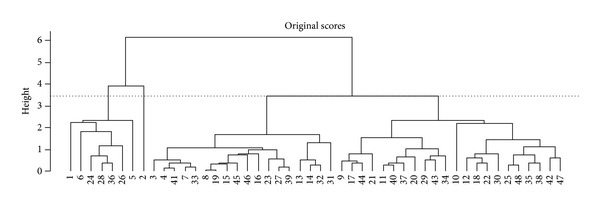
Dendrogram for average AHA; original PC scores as input. Two clusters and an outlier are suggested at height = 3.47.

**Figure 6:**
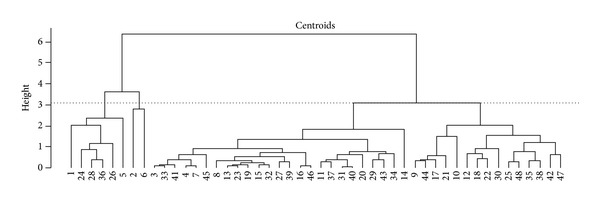
Dendrogram for average AHA; BST centroids as input. Three clusters are suggested at height = 3.07.

Scores from the original first principal plane were used as inputs for the AHA. According to the chosen separation distance (height), it was possible to identify (at least) two clusters, one with signals 1, 5, 6, 24, 26, 28, and 36 and another with all other signals (Figure 5). Signal number 2 was merged at the highest separated height, suggesting that this signal is an outlier.  Figure 6 shows the Dendrogram obtained from the BST centroids, in which signal numbers 2 and 6 could be considered as another cluster.

## 6. Discussion

Validation is an important step in any statistical model and PCA is not an exception to this rule [3]. In PCA, distances between scores in a sample cannot be supposed to represent unbiasedly the true distances, especially if the sample size is small [35], and, in this context, an analysis of the sampling variability of the scores is important, allowing for smaller inferential errors.

As predicted by Efron and Tibshirani [14], increased processing power made the BST an important tool in statistical inference and model validation [36, 37]. However, when BST is applied to models that incorporate the SVD algorithm, care must be taken, due to reflection, stretching, or rotation of the principal components [4, 19]. To overcome these problems, this paper used “supplementary points” projected onto the space spanned by the principal components through a Partial BST, thus avoiding techniques such as the Procrustes Analysis simplifying the validation analysis [4]. Hence, the nonparametric BST is a reliable tool for result validation, as long as one takes into account the mentioned problems of different vector spaces generated by BST.

As mentioned, the C.I. of the replicated eigenvalues indicated that two PCs had to be retained. Indeed, an overlapping between the second up to the fifth replicated eigenvalues was present (not shown). The studied dataset had two highly positively correlated variables, a common feature when analyzing spectral power and time-domain EEG, and high correlation between variables usually results in a small number of axes to be retained (typically two). Thus, confidence regions were drawn in two dimensions, but, for dimensionalities larger than two, confidence polygons could be easily plotted using the described technique. Additionally, it should be noted that the first PC represented an overall average, a very common situation in biological data.

Agglomerative Hierarchical Algorithms are one of the unsupervised methods most used in classification studies [10]. Since PCA is sensitive to outliers, an initial analysis of the first principal plane together with the Dendrogram built from the original scores (Figures 4(a) and 5) would suggest that signal 2 was an outlier. However, this interpretation was not confirmed, since the confidence polygons of signals 2, 24, 28, and 36 had overlapping regions corresponding to condition A, thus suggesting a similarity among them (Figure 4(b)). Furthermore, when the BST centroids were used as input data, signal 2 was first merged with signal 6, suggesting a different group (Figure 6). This feature was not detected when the original scores were used as input data for the classifier.

As mentioned, PCA is widely used in biomedical signal analysis. For example, Casarotto et al. [38] employed PCA for reducing ocular artifacts in event-related potentials (in 39 children) by subtracting the principal component related to the electrooculogram (EOG) from the raw EEG. Since EOG is always present and has amplitude similar to EEG, reducing artifacts from this source is very important, and the authors concluded that the approach allowed for an efficient reduction of ocular artifacts. Kobayashi and Kuriki [39] employed PCA to increase the signal-to-noise ratio (SNR) in evoked neuromagnetic signals applied to four male subjects. The raw spontaneous neuromagnetic fields were recorded by a superconducting quantum interference device (SQUID) system and superposed to simulated evoked fields to mimic real signals. The authors retained three PCs for analysis and concluded that the suppression of the first PC improved the SNR compared to the common averaging method. Also Daffertshofer et al. [40], analyzing six electromyographic (EMG) signals from thoracic and lumbar muscles, obtained during a treadmill walking experiment, found that the first two PCs accounted for 88% of the data variance. The second PC suggested a contrast between right and left thoracic muscles, while the first PC represented an overall average. Analysis of gait kinematic data in stroke patients was performed in twenty-seven subjects by Milovanovic and Popović [41] who found differences between patients and healthy subjects by PCA. In that study, the authors retained the first two principal components and concluded that the first PC is related to severity of hemiplegia.

However, none of the studies above included a discussion about the generalization potential of their results. The methodology described here would be very useful to this end, owing to the small number of subjects in many of these studies. Furthermore, since PC scores can be used as input data in classification algorithms for BCI purposes, this assessment is especially important for avoiding inaccurate analysis in the training dataset.

The results suggested two and three main clusters for the analyzed dataset, mainly due to the importance of the first PC. As it is well-known, the occipital area (O1 and O2 derivations) is recognized as a visual area in the human cortex, while the parietal area (P3 and P4) is known to be part of the associative cortex, which corresponds to the sensory-motor integration within postural control. When individuals are in standing up position (orthostatic posture), especially in the eyes closed condition, other sensory (vestibular and proprioceptors) systems play an important role in balance, increasing the activity in other cortex regions [42]. Therefore, we analyzed only stable postural conditions in which the volunteers were kept in “sat” position, to minimize the influence of other EEG derivations. Usually the cortical activity during balance perturbation is investigated in time domain by the coherence average method, to analyze the latency of the evoked potential after stimulation onset [43]. However, the evoked potential evaluation is not an automatic process of stimuli response identification, and objective response detection (ORD) techniques in frequency domain, such as the spectral *F*-test (SFT) and the event-related desynchronization/synchronization index (ERD/ERS), have been used to this end [44, 45].

In summary, this paper showed how BST methods can be applied to validate the most important PCA results, what is particularly relevant in small data sets, a common feature in EEG studies. One of the presented methods is a new procedure, which consists in estimating new PC scores as centroids of confidence regions calculated by a PBST of the original data (the BST centroids) and in using these centroids as a validation set. A comparison was performed on two Agglomerative Hierarchical Algorithms, one with the original and the other using the estimated component scores as inputs, and the estimated scores allowed for the detection of a cluster not discovered by the original scores. Furthermore, the confidence regions were able to help result interpretation, for instance, by the analysis of their overlap. As discussed, in this case, the area of a polygon increases together with the variability of the PC, providing additional insights about the data, for instance, concerning outliers and remote observations in the multidimensional space [46]. Studies using more complex classification algorithms and data with dimensionality larger than two would be useful for further developing this work.

## Figures and Tables

**Table 1:** Loadings matrix showing the coefficients for the first two PCs in a PCA of 10 variables, relating to EEG signals from 24 volunteers.

Variables	1° PC	2° PC
Alpha power	−0.33	0.10
Beta power	−0.29	** 0.57**
Theta power	−0.31	−**0.38**
Alpha max.	−0.32	0.10
Beta max.	−0.30	** 0.51**
Theta max.	−0.30	−**0.43**
RMS	−0.33	−0.09
SD	−0.33	−0.09
Skewness	−0.29	−0.21
Mm	−0.34	−0.06
